# No evidence for paradoxical effects of tocilizumab in rodents

**DOI:** 10.1007/s00210-025-04021-1

**Published:** 2025-03-12

**Authors:** Christoph Garbers

**Affiliations:** Institute of Clinical Biochemistry, Hannover Medical School, Carl-Neuberg-Strasse 1, 30625 Hannover, Germany

**Keywords:** Interleukin-6, Tocilizumab, PI3K/Akt signaling

## Abstract

Interleukin-6 (IL-6) is a multifunctional cytokine with important functions in health and disease. In order to activate its target cells, IL-6 binds first to the IL-6 receptor (IL-6R), which in turn induces the recruitment and homodimerization of the signal-transducing β-receptor gp130 and the activation of intracellular signaling cascades, including the phosphoinositide 3-kinase (PI3K)-AKT cascade. IL-6 is involved in the pathogenesis of multiple inflammatory diseases, and tocilizumab, a monoclonal antibody that binds to the IL-6R and thus blocks the biological activities of IL-6, is in clinical use worldwide for the treatment of patients with inflammatory diseases, including rheumatoid arthritis. Recently, Weng and colleagues published a paper in *Naunyn–Schmiedeberg’s Archives of Pharmacology* describing paradoxical effects of tocilizumab when used on murine cells in vitro and in a rat model of acute lung injury in vivo. In this communication, I provide evidence that the results presented by Weng and colleagues are not compatible with what is known about the biology of IL-6 and highlight why the provided evidence is insufficient to believe that tocilizumab shows the reported paradoxical effects in rodents.

Interleukin-6 (IL-6) is a multifunctional cytokine with important functions in health and disease. Because IL-6 serum levels are low in healthy individuals, but highly upregulated in basically all inflammatory diseases, IL-6 has been recognized more than 20 years ago as a suitable therapeutic target. Since then, several antibodies, designer proteins, and small compounds have been developed that target individual proteins of the IL-6/gp130/STAT3 signaling axis to inhibit IL-6-mediated signaling (Garbers et al. 2018). The first approved antibody was tocilizumab, a humanized monoclonal antibody that binds to the IL-6R and thereby prevents binding of IL-6 and thus the initiation of the whole signaling cascade.

In contrast to several other cytokines, IL-6 has species-specific properties. Human IL-6 can bind the human and the murine IL-6R, while murine IL-6 can of course bind to the murine IL-6R but has no affinity for the human IL-6R (Coulie et al. 1989; van Dam et al. 1993). This can be explained due to important structural features in which human and murine IL-6R differ (Wiesinger et al. 2009) and due to the fact that the so-called *site I*, which mediates the direct interaction between IL-6 and the IL-6R, is not conserved between human and murine IL-6R (Lokau et al. 2020). Accordingly, several studies have shown that tocilizumab is not able to block IL-6 signaling through the murine or the rat IL-6R (Nishimoto et al. 2000; Okazaki et al. 2002; Garbers et al. 2011; Lokau et al. 2020; George et al. 2021). This is further underlined by the fact that pre-clinical studies in rodents, which were required for the approval of tocilizumab for use in human patients, could not be performed with tocilizumab. They had to be done instead with a surrogate antibody called MR16-1 that blocks murine and rat IL-6R (EMA n.d). We had compared the action of tocilizumab on human and murine cells several years ago and found, in line with several other studies, no evidence for the inhibition of tocilizumab on murine cells (Lokau et al. 2020). Analysis of tocilizumab in mice is only possible in genetically modified mice in which the endogenous gene locus encoding the murine IL-6R is replaced by the human *IL6R* gene (Ueda et al. 2013).

I was therefore surprised to read a paper published recently in *Naunyn–Schmiedeberg’s Archives of Pharmacology* by Weng and colleagues that reported paradoxical effects of tocilizumab in lipopolysaccharide‑induced acute lung injury using a murine cell line in vitro and an animal model with rats in vivo (Weng et al. 2025). As described above, the published evidence shows that tocilizumab can neither bind to the IL-6R present on the murine cell line nor to the IL-6R present in the rats. Contradictory data showing that tocilizumab is indeed effective in rats or murine cells is not presented in the publication by Weng et al. Thus, it is not possible that the reported effects stem from successful inhibition of IL-6 signaling.

Besides this, the paper presents other results that are incompatible with what is known about the biology of IL-6. The authors induce the acute lung injury by injection of LPS, a known activator of NF-κB-mediated gene transcription, which results in the induction of pro-inflammatory cytokines like IL-1β, TNFα, and IL-6, as shown in Fig. 4 of their manuscript (Weng et al. 2025). Surprisingly, the authors report a de-activation of PI3K/Akt signaling, which was shown by reduced phosphorylation of PI3K and Akt in Fig. 1 of their manuscript. As mentioned above, IL-6 activates PI3K/Akt signaling after binding to the IL-6R, which would result in increased phosphorylation of PI3K and Akt (Eulenfeld et al. 2012) (Fig. 1A). Similarly, both TNFα and IL-1β are known as strong activators and not repressors of PI3K/Akt signaling (see, for example, Xu et al. (2022), Tabei and Nakajima (2024)). Furthermore, the authors report in the same figure an increase in phosphorylation of PI3K and Akt when tocilizumab is given to the animals. Blockade of the IL-6R would reduce and not increase the activation of PI3K/Akt signaling (see, for example, Mochizuki et al. (2015)) (Fig. 1B). A similar problem occurs in Fig. 2 of their paper, which reports basically the same findings in the mouse cell line in vitro. However, stimulation of cells with LPS would result in direct activation of PI3K/Akt signaling even without the involvement of IL-6 and other cytokines, as shown more than two decades ago (see, for example, Monick et al. (2001)). The reported reduction in pAkt and pPI3K is therefore not plausible, and why blockade of the IL-6R should influence LPS-mediated signaling, which is sensed by TLR4 and not IL-6R, is also unclear.

**Fig. 1:**
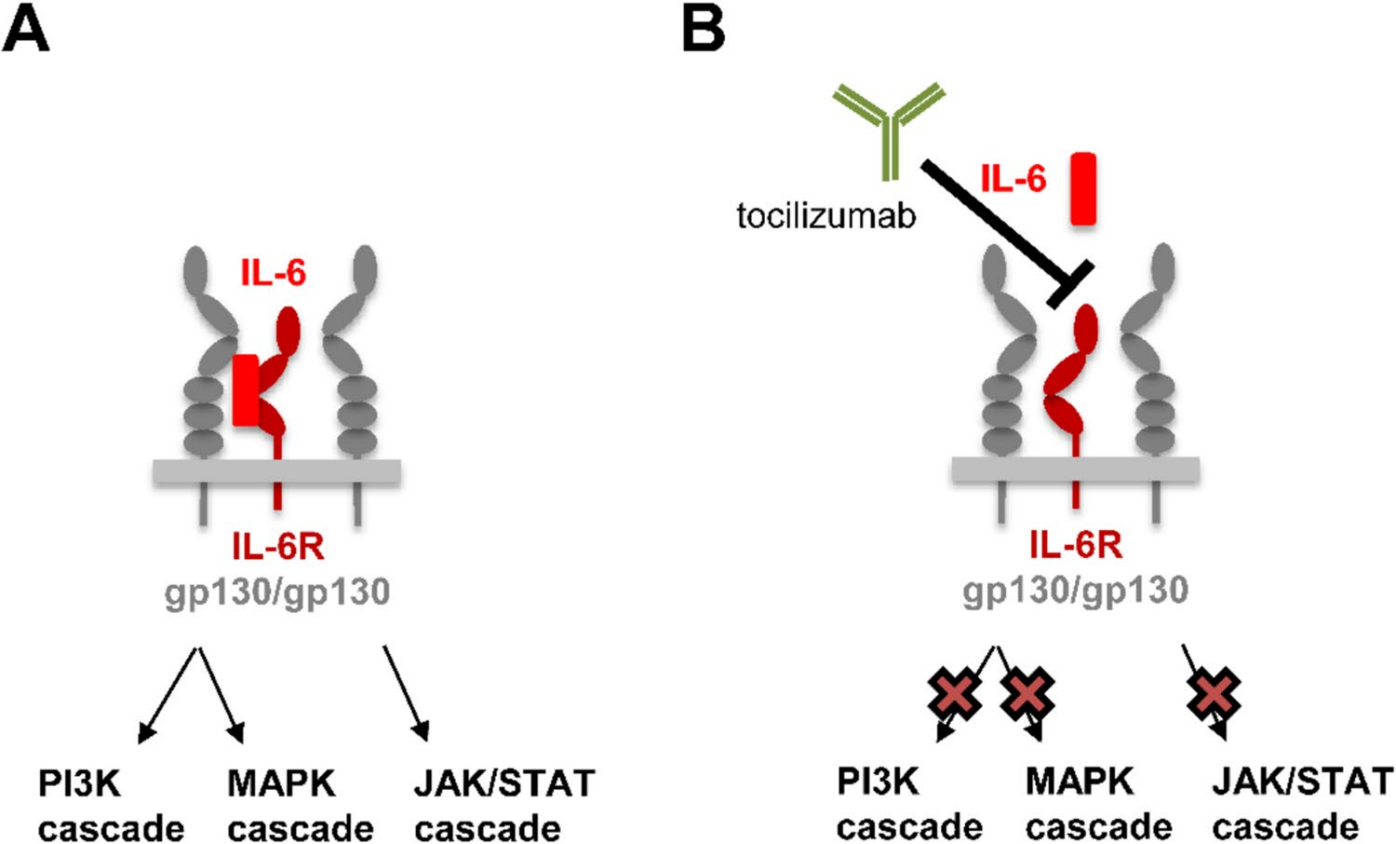
The signal transduction complex of IL-6 and its inhibition by tocilizumab. **A** IL-6 (red) binds to the IL-6R (dark red) on its target cells. The formation of the initial IL-6/IL-6R complex triggers the recruitment and formation of a homodimer of the signal-transducing β-receptor gp130, followed by the activation of the intracellular signaling cascades PI3K, MAPK, and JAK/STAT. **B** The monoclonal antibody tocilizumab binds to the IL-6R, thereby preventing binding of IL-6 to the IL-6R and inhibiting the activation of the intracellular signaling cascades

Another point of concern are the reported pulmonary levels of IL-6 in the animals, which are significantly higher in animals receiving tocilizumab in combination with the ERK inhibitor LY294002 compared to animals treated with tocilizumab only (Fig. 4C of Weng et al. (2025)). IL-6 is removed from the circulation through binding to the IL-6R and the subsequent internalization of the IL-6/IL-6R/gp130 signaling complex (Flynn et al. 2021). Blockade of the IL-6R by tocilizumab would prevent this, and indeed patients treated with tocilizumab show increased IL-6 levels in the circulation due to the lack of IL-6 uptake into the tissues (Nishimoto et al. 2008; Shimamoto et al. 2013). If the IL-6R in the rats would successful be blocked by tocilizumab, one would expect similar IL-6 levels in the lungs of the two animal groups, because inhibition of ERK plays no role in binding of IL-6 to the IL-6R. The reported differences in Weng et al. underline that tocilizumab does not work as intended when injected into rats.

In their discussion, the authors argue that a reduction of pulmonary cytokine levels is indicative of the anti-inflammatory potential of tocilizumab and that “by inhibiting IL-6 signaling, [tocilizumab] effectively reduced the levels of pro-inflammatory cytokines.” This is a fundamental misunderstanding of the biology of IL-6. Production and release of IL-6 after an inflammatory insult (in this case LPS) has nothing to do with the biological function of IL-6 after binding to the IL-6R on the target cells. LPS binds to TLR4, and the activated NF-kB transcription factor leads to the transcription of IL-6 mRNA (and of other pro-inflammatory cytokines like TNFα), subsequently resulting in the production of IL-6 protein and its release from the cell into the extracellular space. This cytokine production does not require IL-6R at any step and can be performed by any cell type of the human body, irrespective whether it expresses the IL-6R on its surface or not. Tocilizumab does not interfere with this process at all—tocilizumab only blocks binding of IL-6 on the IL-6R-expressing cells and thereby inhibits its biological functions, but not the initial production of IL-6 (Fig. 1B). Reduced cytokine levels are thus not indicative of successful inhibition of the IL-6R; instead, increased IL-6 levels in the serum of treated animals (which are not reported in the paper by Weng et al.), as seen in treated patients, would be indicative of tocilizumab blocking the IL-6R.

This is not the only oddity in the discussion section, as the authors later claim that “activation of the IL-6 receptor can inhibit the intracellular PI3K/Akt signaling pathway” (Weng et al. 2025), citing two publications as evidence for this assertion. However, Sun et al. (2020) present no data in their paper showing that activation of the IL-6R can inhibit PI3K/Akt signaling, and the paper by Patruno et al. (2012) does not even mention IL-6 or the IL-6R. Thus, the authors do not present any evidence for their claim that activation of the IL-6R inhibits PI3K/Akt signaling, and as I have described above, the exact opposite is true.

As summarized in Table 1, the effects of tocilizumab reported in the paper by Weng et al. are not plausible, are directly contrary to what has been published by several other groups in the last two decades, and are not compatible with what is known about the biology of IL-6.

**Table 1:** Comparison of the main claims and results presented in Weng et al. with what is known in the literature regarding the biology of IL-6

	(Weng et al. 2025)	literature	References
Tocilizumab	Active on murine cells	Inactive on murine cells	(Nishimoto et al. 2000; Okazaki et al. 2002; Garbers et al. 2011; Lokau et al. 2020)
Tocilizumab	Active when injected in rats	Inactive when injected in rats	(Okazaki et al. 2002; George et al. 2021)
Effect of IL-6 on PI3K/Akt signaling	Inactivation	Activation	(Eulenfeld et al. 2012)
Effect of tocilizumab on PI3K/Akt signaling	Activation	Inactivation	(Eulenfeld et al. 2012; Mochizuki et al. 2015)
Pulmonary levels of IL-6	Increased when rats are treated with tocilizumab and ERK inhibitor	Independent of ERK; should be reduced when IL-6R is blocked	(Nishimoto et al. 2008; Shimamoto et al. 2013; Flynn et al. 2021)

## Data Availability

No datasets were generated or analysed during the current study.
